# Metabolic dysregulation and decreased capillarization in skeletal muscles of male adolescent offspring rats exposed to gestational intermittent hypoxia

**DOI:** 10.3389/fphys.2023.1067683

**Published:** 2023-01-12

**Authors:** Wirongrong Wongkitikamjorn, Eiji Wada, Jun Hosomichi, Hideyuki Maeda, Sirichom Satrawaha, Haixin Hong, Ken-ichi Yoshida, Takashi Ono, Yukiko K. Hayashi

**Affiliations:** ^1^ Department of Orthodontic Science, Graduate School of Medical and Dental Sciences, Tokyo Medical and Dental University (TMDU), Tokyo, Japan; ^2^ Department of Orthodontics, Faculty of Dentistry, Chulalongkorn University, Bangkok, Thailand; ^3^ Department of Pathophysiology, Tokyo Medical University, Tokyo, Japan; ^4^ Department of Forensic Medicine, Tokyo Medical University, Tokyo, Japan; ^5^ Department of Stomatology, Shenzhen University General Hospital, Shenzhen, China

**Keywords:** gestational intermittent hypoxia, skeletal muscle, developmental origins of health and disease (DOHaD), energy metabolism, adiponectin receptors, capillarization

## Abstract

Gestational intermittent hypoxia (IH) is a hallmark of obstructive sleep apnea that occurs frequently during pregnancy, and effects caused by this environmental change during pregnancy may be transmitted to the offspring. In this study, we aimed to clarify the effects of IH in pregnant rats on the skeletal muscle of adolescent offspring rats. Mother rats underwent IH from gestation day 7–21, and their 5-weeks-old male offspring were analyzed. All male offspring rats were born and raised under normoxia conditions. Although no general growth retardation was observed, we found that exposure to gestational IH reduces endurance running capacity of adolescent offspring rats. Both a respiratory muscle (diaphragm; DIA) and a limb muscle (tibialis anterior; TA) showed no histological abnormalities, including fiber size and fiber type distribution. To identify the possible mechanism underlying the reduced running capacity, regulatory factors associated with energy metabolism were analyzed in different parts of skeletal muscles. Compared with rats born under conditions of gestational normoxia, gestational IH offspring rats showed significantly lower expression of genes associated with glucose and lipid metabolism, and lower protein levels of phosphorylated AMPK and AKT. Furthermore, gene expression of adiponectin receptors one and two was significantly decreased in the DIA and TA muscles. In addition, the DIA muscle from adolescent rats had significantly decreased capillary density as a result of gestational IH. However, these changes were not observed in a sucking muscle (geniohyoid) and a masticating muscle (masseter) of these rats. These results suggest that respiratory and limb muscles are vulnerable to gestational IH, which induces altered energy metabolism with decreased aerobic motor function. These changes were partially owing to the decreased expression of adiponectin receptors and decreased capillary density in adolescent offspring rats.

## 1 Introduction

Obstructive sleep apnea (OSA) is prolonged partial and/or intermittent complete airway obstruction during sleep (ATS American Thoracic Society, 1996), which consequently leads to intermittent hypoxia (IH) (Dewan et al., 2015). The prevalence of OSA in the third trimester of pregnancy is as high as 15.4% (Louis et al., 2012), owing to reduced upper airway dimensions, partially caused by pharyngeal edema (Izci et al., 2006) and pregnancy rhinitis (Izci Balserak, 2015). The categorization of the origin of fetal hypoxia by Kingdom and Kaufmann states that OSA during pregnancy causes preplacental hypoxia, which leads to a lower fetal partial pressure of oxygen (Nicolaides et al., 1987; Kingdom and Kaufmann, 1997). Together with anaerobic glycolysis, oxidative phosphorylation in mitochondria is required for the increasing energy demand during fetal development (Baker and Ebert, 2013). The concept that the environmental factors of a fetus influence the organism’s adaptation to conditions later in life is known as the Developmental Origins of Health and Disease paradigm (DOHaD) (Heindel et al., 2015). Several studies have demonstrated that gestational IH causes health concerns in an organism later in life, such as diabetes mellitus, hypertension, and cardiovascular diseases (Hutter et al., 2010; Giussani et al., 2012; Svitok et al., 2016; Badran et al., 2019).

In recent years, animal models of prenatal hypoxia have been widely used to understand the molecular mechanisms of adverse outcomes in offspring. Prenatal hypoxia affects fetal growth and elicits many disturbances after birth including the development of the central nervous system and cardiovascular regulatory system (Peyronnet et al., 2000; Wang et al., 2021; Sutovska et al., 2022). Decreased oxygen supply and peripheral blood flow by gestational IH to the fetal organs such as the heart and brain have critical impacts on physiological functions of these organs (Baschat et al., 1997). In addition, skeletal muscle is not fully developed in the fetus. Skeletal muscle requires to increase the blood flow to meet the substantial increase of oxygen demand from muscle contraction especially during aerobic exercise; however, adverse outcomes of gestational IH on skeletal muscles of the offspring remain unclear.

The effects of postnatal chronic IH exposure in the skeletal muscle of rodents have been previously reported (Shortt et al., 2014), including decreased muscle force and endurance associated with a shift in fiber type from oxidative (slow) to glycolytic (fast) in the diaphragm (DIA) muscle (Shortt et al., 2013), and changing muscle endurance in sternohyoid muscles (upper airway muscles) (Dunleavy et al., 2008). In contrast, gestational IH without postnatal IH exposure did not cause *ex vivo* muscle dysfunction in the diaphragm and sternohyoid muscles in both male and female adult offspring rats (McDonald et al., 2016). Furthermore, we recently showed that gestational IH without postnatal IH exposure induces mitochondrial impairment in a sucking muscle (geniohyoid muscle; GH), but not in a masticating muscle (masseter muscle; MAS) in male adolescent offspring rats (Wongkitikamjorn et al., 2022). These results indicate that skeletal muscles from offspring have site-specific susceptibility to gestational IH. The aim of this study was to clarify the effects of gestational IH on muscle morphology, function, and metabolism in a respiratory muscle (DIA) and a limb muscle (tibialis anterior; TA) in adolescent offspring rats. GH and MAS muscles were also analyzed to compare site-specific differences.

## 2 Materials and methods

### 2.1 Experimental model of IH

IH causes hypoxemia and lower oxygen availability in animals. Various protocols of gestational and postnatal IH exposure were used in previous studies to analyze the effects of the severity of the functional impairments, which were different in oxygen percentage, number of cycles, duration of exposure, and timing (Navarrete-Opazo and Mitchell, 2014). The IH protocol used in this study has been described previously (Nagai et al., 2014; Hong et al., 2021). Briefly, 7-weeks-olds rats were exposed to 4% oxygen every 3 min periods for 8 h/day for 14 days to evaluate autophagic regulation in cardiac muscle (Maeda et al., 2013). In this study, Sprague-Dawley rats on the seventh day of pregnancy were randomly divided into the normoxia (*n* = 3) and IH (*n* = 3) groups, and housed under normoxia and IH conditions, respectively, for 14 days until the time of delivery. Decreased blood oxygen saturation levels were observed using a pulse oximeter (MouseOx; STARR Life Sciences Corp., United States) in pregnant rats in the IH condition, as previously described (Wongkitikamjorn et al., 2022). Offspring rats were born naturally and housed under normoxia with their mothers until weaning at day 21 after birth. All rats were maintained in a specific pathogen-free facility with 12-h/12-h light/dark cycles, and food and water were given *ad libitum*. Gestational normoxia and postnatal normoxia control group is named as N/N, and gestational IH and postnatal normoxia group is named as IH/N. Male offspring rats of each group were weighed every week and euthanized at the age of 5 weeks for further analysis. Food consumption was measured at 25 and 35 days after birth.

All experimental procedures were performed according to the Guide for the Care and Use of Laboratory Animals published by the United States National Institutes of Health (NIH publication 85-23). The Animal Care and Use Committee of Tokyo Medical University approved all experiments performed in this study (study approval number: H31-0011).

### 2.2 Grip strength test

Forelimb grip strength was assessed using a grip strength meter (CPM-101B, Melquest, Japan). Rats (5 weeks of age, *n* = 6 in each group) were placed on a grid, and then was pulled backward by their tails until they released the grid. The peak pull force was recorded on a digital force transducer. The test was repeated 3 times for each rat, and the interval of each test was 30 s. The averages of the repeated grip strength measurements were normalized to body weights.

### 2.3 Exhaustion treadmill running test

Aerobic motor function was evaluated using a treadmill. Rats from both groups (5 weeks of age, *n* = 6 in each group) were familiarized with a motorized treadmill containing shocker plates for 2 days (at 5 m/min for 30 min per day). The protocol used for the exhaustion test was as previously described (Wada et al., 2019). Briefly, the test was started at 5 m/min for 5 min. The speed was gradually increased by 1 m/min every min until the rat could no longer run continuously.

### 2.4 Sample preparation and histological staining

Five-week-old male rats that did not undergo muscle function tests were anesthetized with isoflurane and euthanized. Soon after being sacrificed, blood samples were collected *via* the caudal vena cava, and serum samples were separated by incubation on ice for 2 h, followed by centrifugation at 8,000 rpm for 15 min. Serum total protein, total cholesterol, triglyceride, high-density lipoprotein cholesterol, glucose, and lactate levels were measured using a biochemistry automatic analyzer (model 7180; Hitachi High-Tech, Japan). Serum adiponectin levels were measured using an ELISA kit (Mouse/Rat Adiponectin ELISA-OY kit; Oriental Yeast Co., Japan), in accordance with the manufacturer’s instructions. Muscle samples were collected and frozen immediately with isopentane in liquid nitrogen then stored at −80°C until use. All frozen samples were cut into transverse 10-µm-thick sections using a Leica CM 3050S cryostat, and collected on micro cover glasses (Matsunami, Japan). Each sample was stained with H&E, modified Gomori Trichrome, nicotinamide adenine dinucleotide reductase (NADH), and periodic acid schiff (PAS). For the NADH staining, cryosections were stained with the NADH solution (1.0% nitro blue tetrazolium and .8% beta-NADH in .05 M Tris-HCl buffer) for 30 min at 37°C. Semiquantitative measurements of the intensity of the NADH and PAS staining in each fiber type was analyzed based on previous reports (Scribbans et al., 2014; Cong et al., 2016; White et al., 2016; Giacomello et al., 2020; Zheng et al., 2020; Corona et al., 2022). For fiber type classification, each section was stained with primary antibodies against myosin heavy chains (MHC) as listed in Supplementary Table S1. Alexa Fluor 350 and 488 anti-mouse and 598 anti-rabbit secondary antibodies (1:1,000; Thermo Fisher Scientific) were used for detection. The percentage area of NADH-positive or PAS-stained from each muscle fiber was calculated from serial sections using NIH ImageJ software, and around 40 fibers of each muscle fiber type were counted from the DIA (type I, IIA, IIX/D) and TA (type I, IIA, IIX/D, IIB) samples (total around 240 fibers of each muscle fiber type in six samples). Each fiber was classified as strong-, medium- or weak-stained by the staining intensity.

### 2.5 Analysis of muscle fiber size and fiber type distribution

Transverse 8-µm-thick muscle cryosections were prepared. After blocking with 2% bovine serum albumin in phosphate-buffered saline, each section was stained with primary antibodies against MHCs and laminin to detect muscle cell membranes at 37°C for 80 min as previously described (Bloemberg and Quadrilatero, 2012). Primary antibodies used for immunofluorescence staining are listed in Supplementary Table S1. For fiber size analysis, Alexa Fluor 488 anti-mouse and 568 anti-rabbit secondary antibodies (1:1,000; Thermo Fisher Scientific, United States) were used for detection. For fiber type distribution analysis, Alexa Fluor 350 and 488 anti-mouse and 598 anti-rabbit secondary antibodies (1:1,000; Thermo Fisher Scientific) were used for detection. All staining images were acquired using a fluorescence microscope (Zeiss, Germany). The whole image of each section was captured by the IN Cell Analyzer 2200 imaging system for calculating muscle fiber size (diameters in the minor axis) with IN Cell Developer Toolbox software (GE Healthcare, United States). Basal membranes were detected by laminin staining to calculated fiber size, and each MHC-positive fiber was automatically selected by staining intensity. Muscle fiber size was assessed by quantifying the short diameters on the cross-sectional images. More than 3,000 fibers (3,000–10,000) from each sample were used for the quantification (*n* = 5 per group). The fiber size distribution was compared between the N/N and IH/N groups. Fiber type compositions were counted using NIH ImageJ software.

### 2.6 Protein extraction and western blotting

Muscle samples were homogenized in a sample buffer solution (Fujifilm, Japan) comprised of RIPA buffer containing protease inhibitors and phosphatase inhibitors (Roche, Switzerland), then centrifuged at 15,000 rpm at 4°C for 5 min. Supernatants were collected, and total protein concentrations were measured using BioPhotometer (Eppendorf, Germany). Equal amounts of protein for each sample were loaded onto 10%–20% or 15% SDS-PAGE gels (Fujifilm) and blotted onto PVDF membranes by the semi-dry technique using Trans-Blot Turbo system (Bio-Rad, United States). The PVDF membranes were incubated with primary antibodies, followed by incubation with horseradish peroxidase-conjugated secondary antibodies (Thermo Fisher Scientific). The primary antibodies used for Western blotting are listed in Supplementary Table S1. All bands were detected with Clarity Western ECL Substrate (Bio-Rad) and visualized using Image Lab 5.0 system (Bio-Rad). All data were normalized using expression levels of GAPDH and analyzed as relative band intensities using Image Lab 5.0.

### 2.7 Quantitative-PCR analysis

Total RNA was extracted from frozen muscle samples using RNeasy Plus Universal Mini kit (QIAGEN, Germany) in accordance with the instructions provided by the manufacturer. Complementary DNA (cDNA) was synthesized from 1,000 ng of total RNA with Oligo (dT) primers using SuperScript IV VILO Master Mix (Thermo Fisher Scientific) in accordance with the manufacturer’s instructions. Real-time PCR was performed using 10 ng of cDNA template for each gene using an Applied Biosystems QuantStudio3 real-time PCR system (Thermo Fisher Scientific). Primers were chosen for real-time PCR as listed in Supplementary Table S2. All results were normalized using *Actb* (beta-actin), and gene expression levels were calculated by the ΔΔCT method of relative quantification. Data were analyzed as relative messenger RNA expression levels.

### 2.8 Quantification of capillary numbers per muscle area and per myofiber

Capillaries, skeletal muscle area and fiber numbers were counted from muscle sections stained with anti-CD31 (an endothelial cell marker) and anti-laminin antibodies, respectively. Alexa Fluor 488 anti-rabbit and 568 anti-goat secondary antibodies (1:1,000; Thermo Fisher Scientific) with DAPI solution were used for detection. Staining sections were observed using a fluorescence microscope Axio Scope A1 (Zeiss), and four random fields of 200-times magnification from each sample (*n* = 6 per group) were used for the measurements. The skeletal muscle area, number of fibers and capillaries in the field were counted using NIH ImageJ software. Data were analyzed as the capillaries per muscle area and per myofiber number.

### 2.9 Statistical analysis

Data are shown as the mean ± standard deviation (SD), and analyzed using the independent *t*-test. A *p*-value of less than .05 was considered to indicate a statistically significant difference between groups. All statistical analyses were performed using SPSS statistics 28 software (IBM, United States).

## 3 Results

### 3.1 Gestational IH reduces endurance running capacity in offspring rats

**FIGURE 1:**
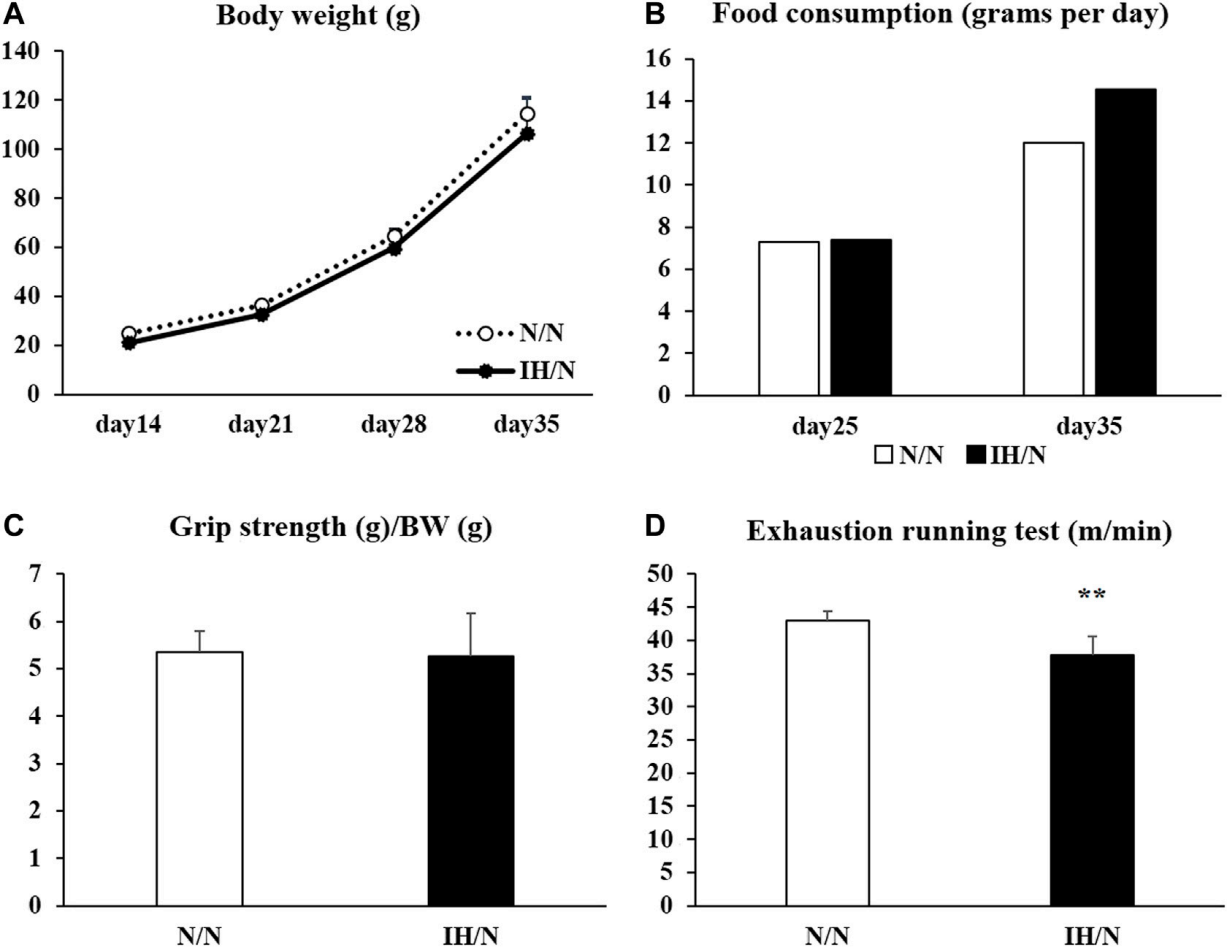
Changes in body weight, food intake, grip strength, and running to exhaustion treadmill test of offspring adolescent rats (N/N and IH/N groups). **(A)** Weekly average body weights of N/N and IH/N rats from day 14 to day 35 after birth were shown. **(B)** Food consumption of N/N and IH/N rats, measured on day 25 and day 35 after birth, was similar. **(C)** Grip strength normalized to body weight showed no significant difference between N/N and IH/N rats on day 35. **(D)** IH/N rats had significantly lower scores on the treadmill running test compared with N/N rats (*n* = 6 in each group) **p < .01.

Both N/N and IH/N offspring rats were born naturally under normoxia, and their body weights gradually increased weekly with no significant difference between the groups (Figure 1A). No general growth retardation was observed in IH/N rats. Food intake was similar in IH/N rats and N/N rats at 25 and 35 days after birth (Figure 1B). Serum biochemical analysis demonstrated that no notable abnormalities were observed, including total cholesterol and triglyceride levels in the IH/N group (Table 1). Serum adiponectin levels were also similar between N/N and IH/N rats.

**TABLE 1:** Serum biochemistry data of N/N and IH/N rats.

	N/N	IH/N	*p*-value
TP (g/dL)	5.88 ± .08	5.82 ± .13	.415
T-CHO (mg/dL)	120.2 ± 13.0	123.0 ± 5.6	.677
TG (mg/dL)	85.4 ± 20.5	102.8 ± 28.2	.301
HDL-C (mg/dL)	44.6 ± 3.65	46.4 ± 2.07	.374
GLU (mg/dL)	430.6 ± 109.9	326.2 ± 67.9	.114
LA (mg/dL)	112.1 ± 21.3	98.3 ± 24.5	.369
Adiponectin (ng/μl)	8138.4 ± 1713.7	10367.9 ± 2085.0	.100

Values are shown as the mean ± SD (*n* = 5 in each group). TP, total protein; T-CHO, total cholesterol; TG, triglyceride; HDL-C, high density lipoprotein cholesterol; GLU, glucose; LA, lactate.

For functional performance tests, the scores of forelimb grip strength (normalized to body weight) showed no significant difference between N/N and IH/N rats (Figure 1C). One day after the grip strength test, maximal exercise performance was evaluated in the rats by treadmill running until exhaustion. When the running speed was gradually increased, IH/N rats stopped running at a slower speed than N/N rats (N/N rats: 43.0 ± 1.4 m/min; IH/N rats: 37.8 ± 2.8 m/min, *p* < .01) (Figure 1D).

### 3.2 Muscle morphology and mitochondria contents are preserved in the DIA and TA from IH/N rats

**FIGURE 2:**
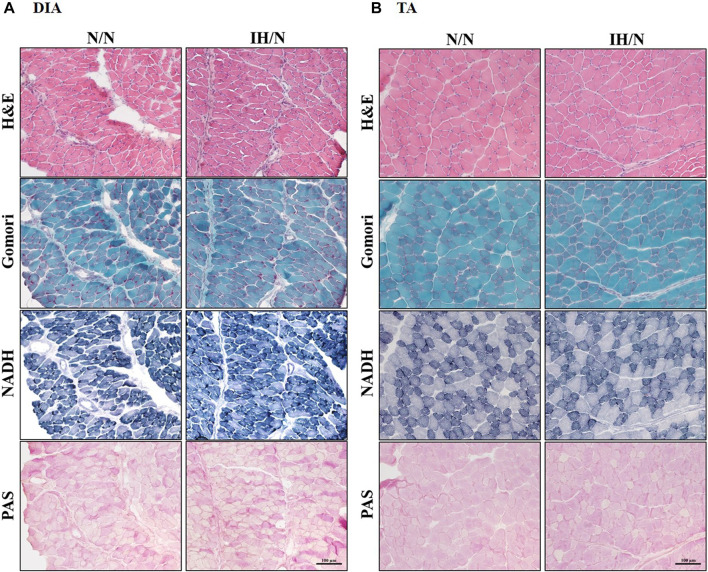
Histological images from the DIA and TA muscles of offspring rats. Histological images of H&E, modified Gomori Trichrome, NADH, and PAS staining showed no pathological features in the **(A)** DIA, and **(B)** TA muscles from N/N and IH/N rats.

**FIGURE 3:**
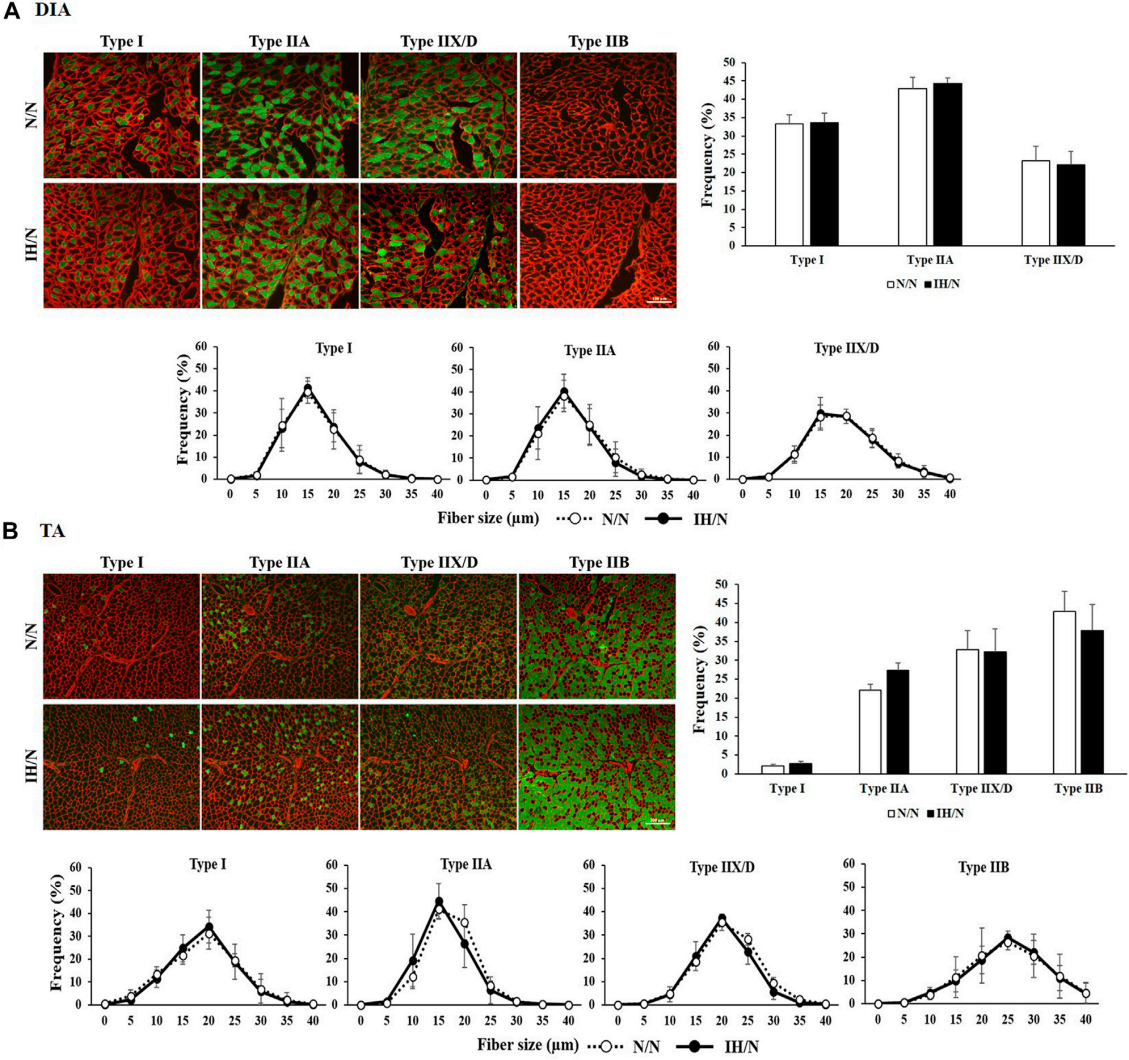
Muscle fiber type distribution and muscle fiber size in the DIA and TA muscles of offspring rats. Fiber type-specific immunohistochemical staining for type I, type IIA, type IIX/D, and type IIB fibers with skeletal muscle membrane protein, laminin (red). Each panel shows the cross-sectional image of the **(A)** DIA, and **(B)** TA muscles. Green areas indicate immuno-positive muscle fibers. Muscle fiber type distribution and size frequency in the DIA and TA showed no significant difference between N/N and IH/N rats. Bars represent **(A)** 100 µm for DIA sections, and **(B)** 200 µm for TA sections.

No pathological changes, such as muscle fiber degeneration, internal nuclei, or inflammatory cellular infiltration were identified by H&E and modified Gomori Trichrome staining in both DIA and TA muscles from N/N and IH/N rats (Figures 2A, B). In addition, there is no notable change in the intensity (classification) of NADH and PAS-stained area of each fiber type in the DIA and TA muscles between N/N and IH/N rats (Figures 2A, B; Supplementary Figures S1A–E). Western blot analysis of mitochondrial biogenesis and fusion proteins indicated that no specific change was observed in the DIA and TA muscles in the adolescent rats exposed to gestational IH (Supplementary Figures S2A–D). In our initial study, gestational IH induced a significant downregulation of mitochondrial proteins in the GH muscle, but not in the MAS muscle (Wongkitikamjorn et al., 2022). The proportion and size distribution of skeletal muscle fiber types showed no significant difference in both DIA and TA muscles from IH/N rats compared with those from N/N rats (Figures 3A, B, respectively). These results indicate that gestational IH affects partly and functionally different skeletal muscles within a body.

### 3.3 Downregulation of genes associated with glucose and lipid metabolism in skeletal muscles from IH/N rats

**FIGURE 4:**
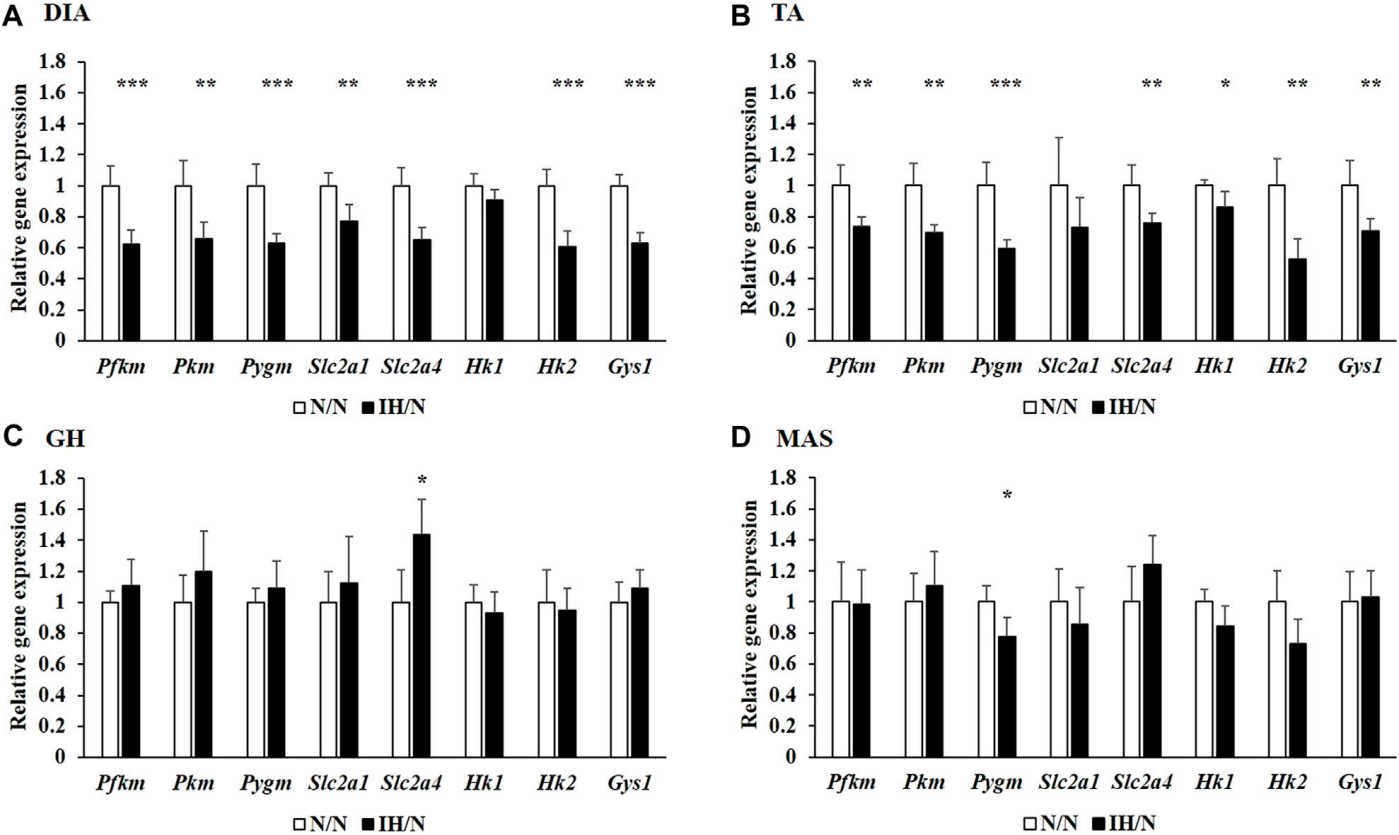
Quantitative PCR analysis of genes associated with glucose metabolism. Relative gene expression of *Pkfm*, *Pkm*, *Pygm*, *Slc2a1*, *Slc2a4*, *Hk1*, *Hk2*, and *Gys1* in the **(A)** DIA, **(B)** TA, **(C)** GH, and **(D)** MAS muscles were normalized by *Actb* and shown as fold increase of the N/N group *p < .05, **p < .01, ***p < .001.

**FIGURE 5:**
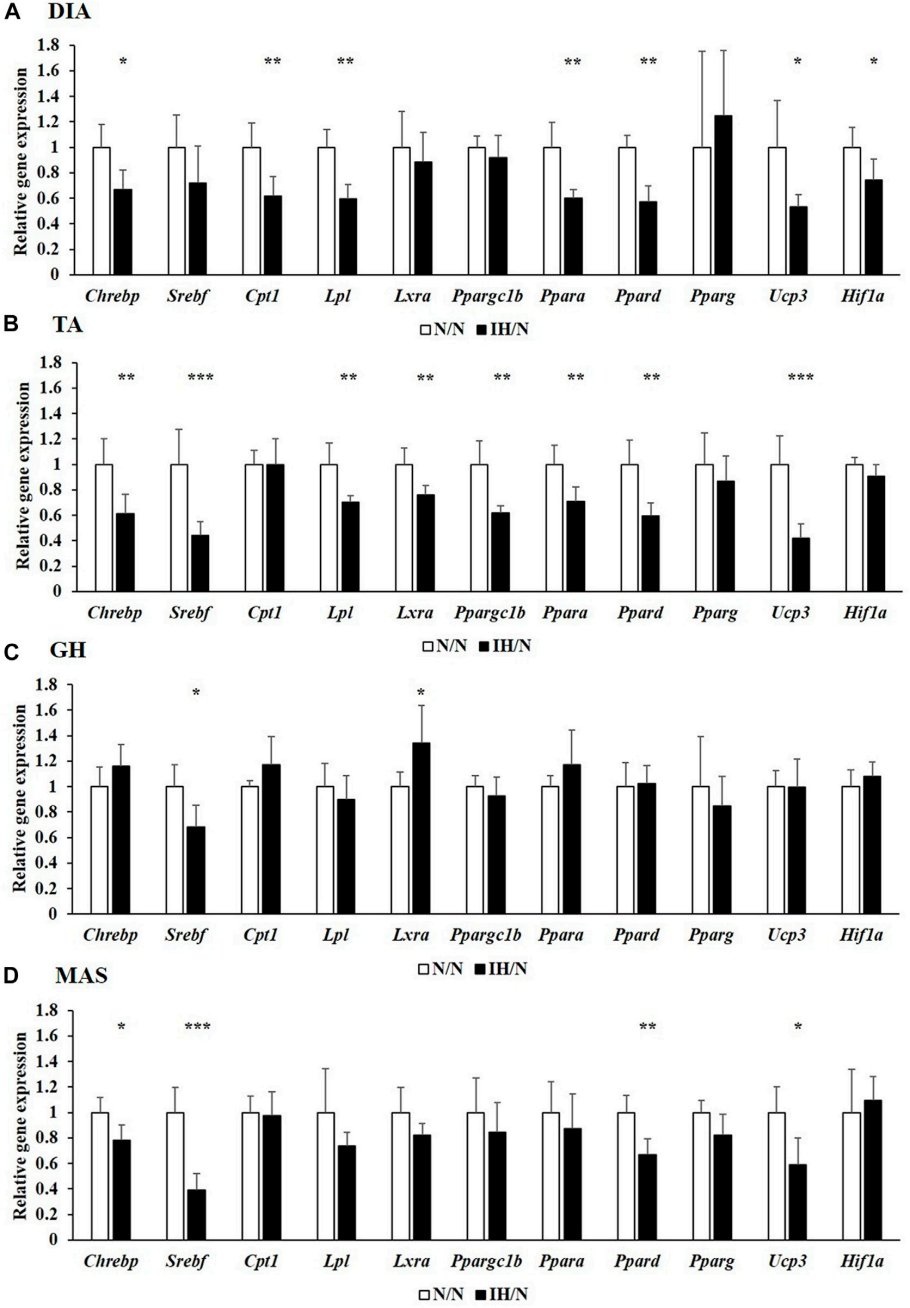
Quantitative PCR analysis of genes associated with lipid metabolism. Relative gene expression of *Chrebp*, *Srebf*, *Cpt1*, *Lpl*, *Lxra*, *Ppargc1b*, *Ppara*, *Ppard*, *Pparg*, *Ucp3*, and *Hif1a* in the **(A)** DIA, **(B)** TA, **(C)** GH, and **(D)** MAS muscles were normalized by *Actb* and shown as fold increase of the N/N group *p < .05, **p < .01, ***p < .001.

Metabolic changes in skeletal muscles in response to gestational IH were assessed by analyzing their relative gene expression changes. The expression of several genes involved in glucose and fatty acid metabolism were downregulated, particularly in the DIA and TA muscles. Among the genes associated with glucose metabolism, the expression of *Slc2a4*, which encodes muscle-enriched glucose transporter 4 (GLUT4), was significantly reduced in the DIA and TA muscles but not in the GH and MAS muscles from IH/N rats (Figures 4A–D). Gene expression levels of *Slc2a1* for GLUT1 and its positive regulator *Hif1a* were also decreased only in the DIA muscle. The expression of glucose metabolic enzymes (*Hk2*, *Pkfm*, *Pkm*, and *Gys1*) were substantially decreased only in the DIA and TA muscles from IH/N rats, and *Pygm* and *Chrebp*, a transcriptional regulator of *Pygm*, were similarly decreased in the DIA, TA, and MAS muscles, but not in the GH muscle from IH/N rats (Figures 4A–D). The expression of several genes involved in fatty acid metabolism was also downregulated particularly in the DIA and TA muscles. Gestational IH reduced the gene expression of triglyceride metabolism (*Lpl*) and fatty acid metabolism (*Ppara*, *Ppard,* and *Ucp3*) in the DIA and TA muscles. In the DIA muscle, a gene associated with beta-oxidation (*Cpt1*) was significantly decreased by gestational IH. Additionally, genes associated with sterol metabolism (*Srebf*) and lipid metabolism (*Lxra* and *Ppargc1b*) were significantly downregulated in the TA muscle from IH/N rats. The expression level of *Srebf* was also commonly decreased in the GH and MAS muscles, whereas *Ppard* and *Ucp3* were significantly decreased in the MAS muscle but not in the GH muscle from IH/N rats (Figures 5A–D). Therefore, alterations in glucose and fatty acid metabolism were substantial in the DIA and TA muscles, but minimal in the GH and MAS muscles.

### 3.4 Suppression of AMPK and AKT activation in the DIA and TA muscles by gestational IH

**FIGURE 6:**
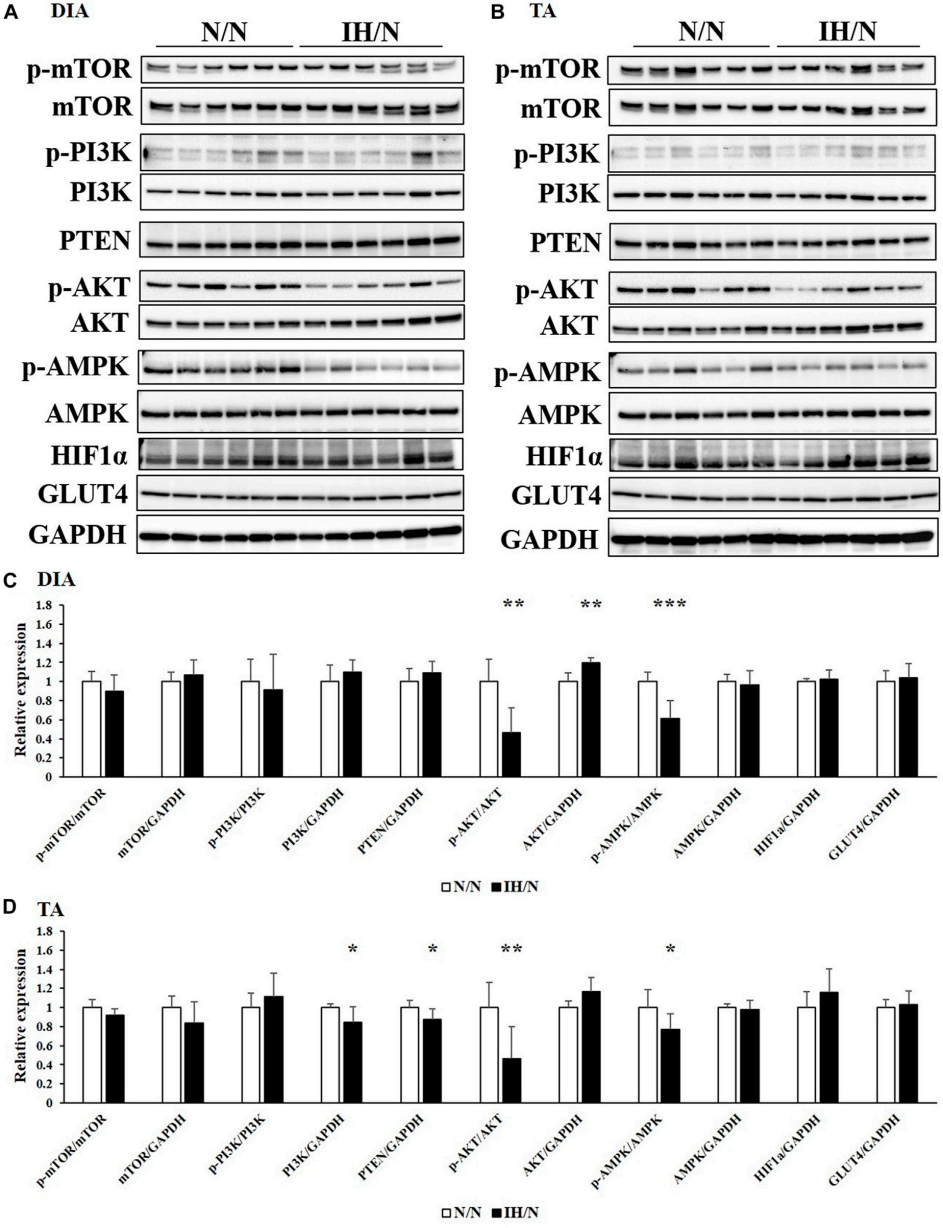
Western blot analysis of proteins involved in glucose and lipid metabolism in the DIA and TA muscles. Western blots were performed on six individual samples to quantify the levels of mTOR, PI3K, AKT, AMPK, PTEN, and HIF1α in the **(A)** DIA, and **(B)** TA muscles. Graphs represent the ratio between the phosphorylated forms of mTOR, PI3K, AKT, and AMPK, and the total amount of each target protein. Relative expression levels of each protein were normalized to the level of GAPDH expression. Expression levels are shown with those for **(C)** DIA, and **(D)** TA muscles from the N/N group set to 1 *p < .05, **p < .01, ***p < .001.

**FIGURE 7:**
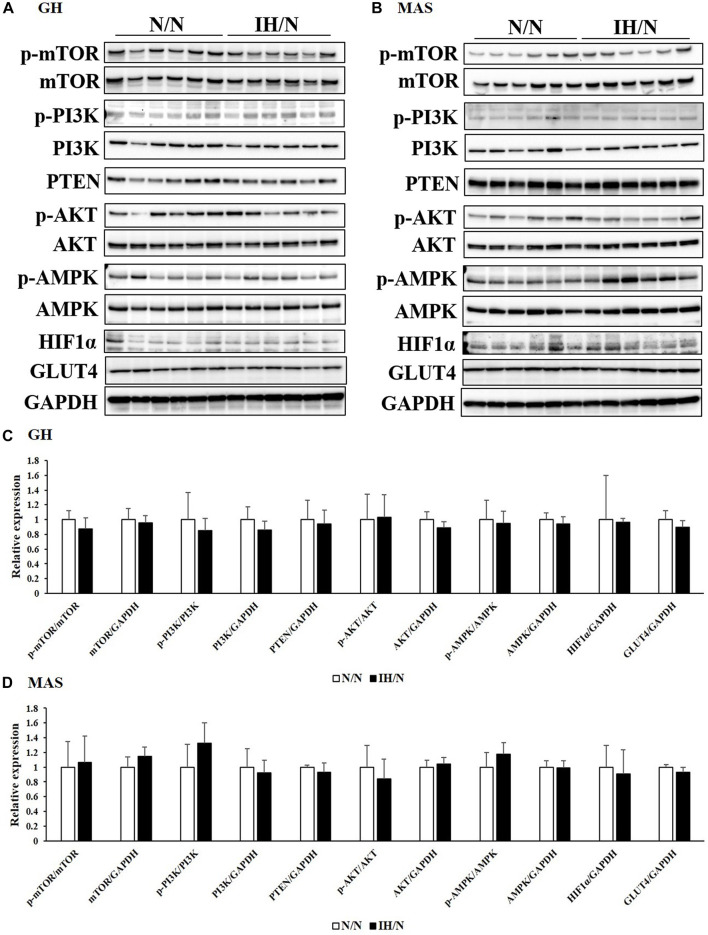
Western blot analysis of proteins involved in glucose and lipid metabolism in the GH and MAS muscles. Western blots were performed on six individual samples to quantify the levels of mTOR, PI3K, AKT, AMPK, PTEN, and HIF1α in the **(A)** GH, and **(B)** MAS muscles. Graphs represent the ratio between the phosphorylated forms of mTOR, PI3K, AKT, and AMPK and the total amount of each target protein. Expression levels of each protein are shown relative to the level of GAPDH expression, for the **(C)** GH, and **(D)** MAS muscles from the N/N group set to 1.

Glucose and/or lipid metabolism is partially regulated by the AMP-activated protein kinase (AMPK) and phosphatidylinositol-3 kinase (PI3K)/protein kinase B (AKT) signaling pathways. The phosphorylation of AMPK, PI3K, AKT, and mammalian target of rapamycin (mTOR), and the expression of associated proteins were analyzed by Western blotting. In the DIA muscle, a significant decrease in AMPK and AKT phosphorylation and increase in total AKT levels were detected (Figures 6A, C). Reduced levels of phosphorylated AMPK and AKT in the TA muscle were also observed (Figures 6B, D). Phosphatase and tensine homolog (PTEN) and total PI3K levels were significantly decreased only in the TA muscle. The expression levels of these proteins were comparable in both the GH and MAS muscles from IH/N rats (Figures 7A–D). Although gene expression levels of *Slc2a4* and *Hif1a* were decreased, the protein levels of GLUT4 and HIF1α were unchanged by gestational IH in all the analyzed skeletal muscles from adolescent rats.

### 3.5 Decreased gene expression levels of adiponectin receptors and capillaries per muscle area and per myofiber

**FIGURE 8:**
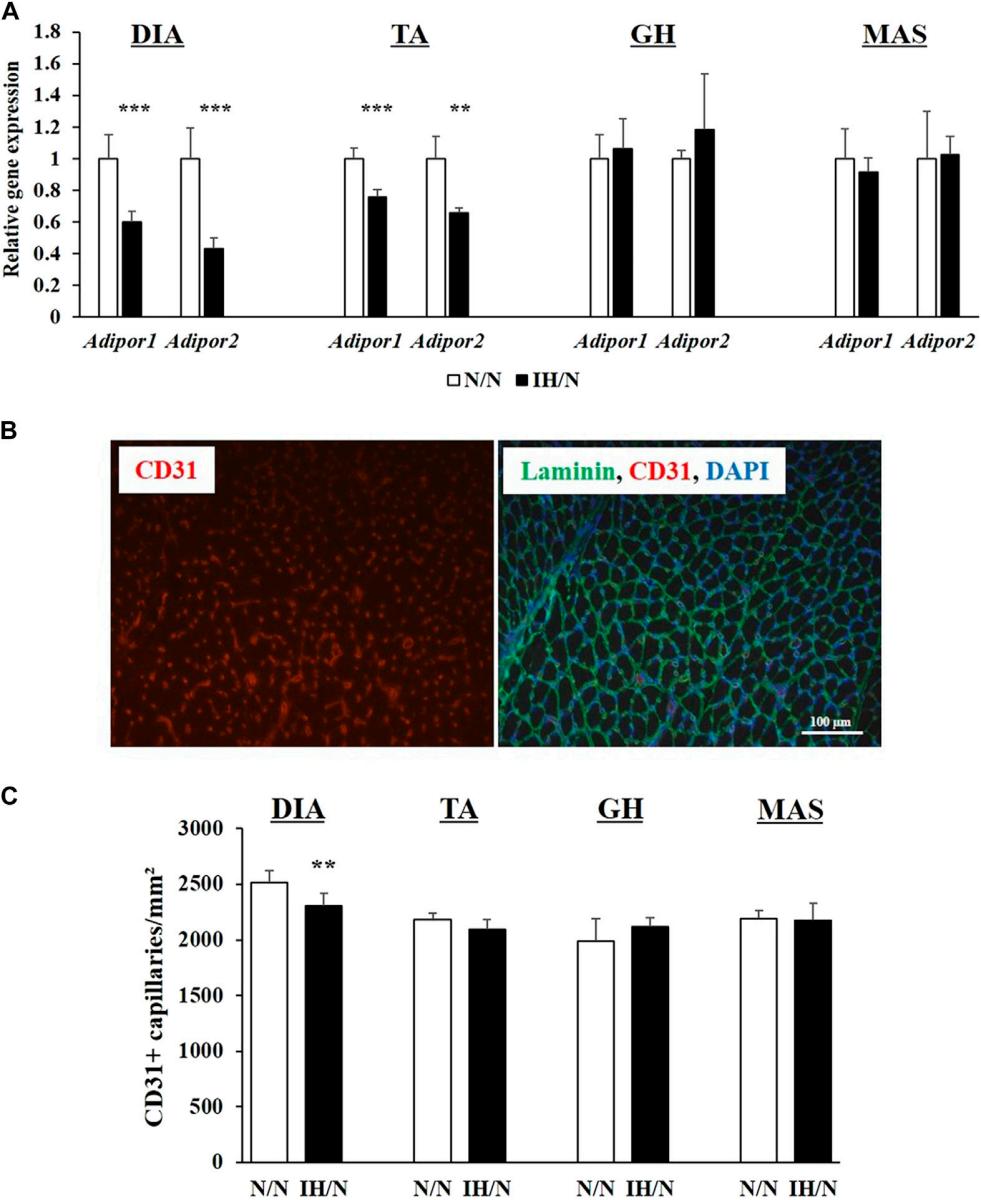
Quantitative PCR analysis of genes encoding adiponectin receptors, and quantification of capillary numbers per muscle area (mm^2^). **(A)** Relative gene expression of *Adipor1* and *Apipor2* in the DIA, TA, GH, and MAS muscles, normalized by *Actb* and shown as fold increase of the N/N group. **(B)** Immunohistochemical staining for CD31 (red) and a merged image with laminin (green) and DAPI (blue) from the TA muscle. Bar represents 100 µm. **(C)** Number of capillaries per muscle area (mm^2^). Results are presented as mean ± SD (*n* = 6) **p < .01, ***p < .001.

Adiponectin, which is an adipocyte-derived circulating hormone, is known to activate AMPK *via* adiponectin receptors, and to improve the utilization of glucose and fatty acids in skeletal muscles (Yamauchi et al., 2002). In skeletal muscle, two adiponectin receptors (AdipoR1 and AdipoR2) are expressed. These two receptors have distinct roles; i.e., adiponectin receptor 1 controls metabolic activity, and adiponectin receptor 2 is associated with vascular homeostasis (Parker-Duffen et al., 2014). Expression levels of both *Adipor1* and *Adipor2* genes were significantly decreased in the DIA and TA muscles, but not in the GH and MAS muscles by gestational IH (Figure 8A). As energy metabolism and blood flow to the skeletal muscle are closely associated with each other, the capillary density in each muscle was calculated as capillaries per muscle area and as a ratio of capillaries-to-myofiber. IH/N rats were found to have decreased capillary per muscle area in the DIA (*p* < .01) and TA (*p* = .07); however, capillarization was not altered in the GH and MAS muscles (Figures 8B, C). These data were consistent with the quantification of capillary numbers per myofiber ratio (Supplementary Figure S3). These results suggested that there is an interaction between reduced *Adipor2* levels and capillary density in the DIA and TA muscles from adolescent offspring rats exposed to gestational IH.

## 4 Discussion

To date, only a limited number of studies have reported the effects of gestational IH on the offspring’s skeletal muscle. In this study, we clearly demonstrated that gestational IH results in a significant reduction in endurance motor function in male adolescent offspring rats, associated with potential metabolic alterations and reduced capillary density in the DIA and TA muscles. Previous studies reported that gestational IH leads to reduced body weight of the offspring at birth, but promotes catch-up growth after birth. Subsequently, adult offspring rats exposed to gestational IH gradually increased their body weight (Gozal et al., 2003; Camm et al., 2011). Likewise, increases in food intake and body weight, together with metabolic abnormalities are observed in an age-dependent manner in adult offspring mice exposed to gestational IH (Khalyfa et al., 2017; Badran et al., 2019). Interestingly, these postnatal changes are sex-dependent. Only male offspring mice are affected, whereas adult female offspring have no metabolic dysfunctions (Badran et al., 2019). Males can be more vulnerable to maternal insults (Dearden and Balthasar, 2014; Sundrani et al., 2017), and therefore, only male offspring rats were analyzed in this study.

In our gestational IH model (IH/N), the growth curves and food intake of these rats were similar to those of control (N/N) rats between 2 and 5 weeks after birth. Serum metabolic parameters, including glucose, total cholesterol, and triglyceride showed no differences between N/N and IH/N rats at 5 weeks of age. These results indicate that gestational IH does not cause overt growth or metabolic abnormalities in adolescent offspring rats.

Importantly, the 5-weeks-old offspring male IH/N rats showed normal grip strength, which is an indicator of forelimb strength, but showed significantly reduced aerobic motor performance as assessed by the forced exhaustion running test using the treadmill, which is a reliable method to assess whole-body muscle function (Wada et al., 2019). Skeletal muscle fiber type composition may be changed by physical activity, pathological or stress conditions, and nutritional conditions. Endurance training, low energy availability, and higher body metabolic rates induce skeletal muscle fiber conversion from glycolytic to oxidative fibers (Purohit and Dhawan, 2019). A previous study reported that conversion from fast to slow fiber type was observed in a CoCl_2_-simulated hypoxic environment of muscle cells (Lixin et al., 2019). Similarly, gestational IH causes hypoxemia and lower O_2_ availability in the fetus (Carter, 2015), which is required for energy demand in aerobic metabolism (Baker and Ebert, 2013). Interestingly, as shown in this study, no histological changes were observed in the DIA and TA muscles in rats exposed to IH, and muscle fiber size and fiber-type composition were comparable between N/N and IH/N rats. Mitochondrial impairment was also not detected in the DIA and TA muscles from IH/N rats. Our recent study demonstrated that gestational IH induces smaller type IIA fibers and mitochondrial impairment in a sucking muscle (GH), but not in a masticating muscle (MAS) in the adolescent offspring rats that had been exposed to gestational IH (Wongkitikamjorn et al., 2022). Even though the effects of mitochondrial impairment on muscle function of GH was not determined, these results indicate that gestational IH may exert different effects on different parts of muscles.

To elucidate the pathomechanism of the reduced aerobic performance observed in IH/N rats, we analyzed the expression of genes associated with energy metabolism. We demonstrated significantly reduced expression of several genes associated with glucose and fatty acid metabolism in the DIA and TA muscles, whereas these changes were minimal in the GH and MAS muscles from the same rats. The downregulation of genes associated with glucose and fatty acid metabolism can be explained by a decrease in phosphorylated AMPK and AKT protein levels (Long and Zierath, 2006). Previous studies reported that offspring rodent pups that were exposed to gestational IH can develop alterations in energy metabolism and have an increased risk of insulin resistance in later life (Camm et al., 2011; Rueda-Clausen et al., 2011; Khalyfa et al., 2014; Badran et al., 2019). Badran et al. reported that offspring mice exposed to gestational IH had increased insulin resistance and decreased phosphorylation of AKT in the gastrocnemius muscle (Badran et al., 2019). On the other hand, Camm et al. demonstrated decreased total AKT2 levels but not phosphorylated AKT levels in skeletal muscle (the specific muscle part was not stated) from adult rat offspring exposed to gestational IH (Camm et al., 2011). These results support our data that the effects of gestational IH on activation of the AKT pathway is different among skeletal muscles of the offspring.

AMPK is another key regulator of energy homeostasis, which increases glucose uptake and fatty acid oxidation in skeletal muscle (Merrill et al., 1997). Furthermore, AMPK can facilitate mitochondrial biogenesis in skeletal muscle (Zong et al., 2002). AMPK is activated by ATP depletion upon rapid muscle contraction, hypoxia, or glucose deprivation. It is also activated by adipokines, such as adiponectin and leptin. Khalyfa et al. (2017) reported decreased serum adiponectin levels and increased serum leptin levels in adult male offspring mice exposed to gestational IH. Interestingly, these mice have less locomotor activity and reduced daily energy expenditure compared with controls (Khalyfa et al., 2017). Low adiponectin levels in serum and perivascular adipose tissue associated with hypermethylation of the adiponectin gene promoter were reported in male adult offspring rats exposed to gestational IH (Badran et al., 2019). Adiponectin, an adipocyte-derived circulating hormone, is known to improve the utilization of glucose and fatty acids in skeletal muscles (Yamauchi et al., 2002). Recent studies demonstrated that adiponectin receptors are also expressed in skeletal muscle. Adiponectin mediates specific effects in organs *via* binding to its receptors, AdipoR1 and AdipoR2 (Bjursell et al., 2007). These two receptors have distinct roles in that AdipoR1, which is mainly expressed in skeletal muscle, regulates energy metabolism, where AdipoR2 controls vascular homeostasis (Parker-Duffen et al., 2014). In our study, reduced expression of the gene encoding AdipoR1 in the DIA and TA muscles but not the GH and MAS muscles was observed by exposure to gestational IH. Consistently, decreased expression of genes associated with energy metabolism together with decreased levels of phosphorylated AMPK and AKT were observed in the DIA and TA, but not in the GH and MAS. Moreover, gene expression of AdipoR2 was similarly reduced in the DIA and TA muscles in male adolescent offspring exposed to gestational IH. In a model of hind limb ischemia, AdipoR2 knockout mice showed delayed recovery of blood flow, impaired perfusion and reduced capillary density in the gastrocnemius muscle (Parker-Duffen et al., 2014). Capillary density is strictly regulated in skeletal muscles in human and rodents, and the ratio depends on the muscle part (O'Reilly et al., 2021; Høeg et al., 2009). Consistent with reduced *Adipor2* expression in the DIA and TA muscles, the number of capillaries per muscle area and per myofiber were significantly decreased in the DIA and slightly decreased in the TA muscles of offspring rats exposed to gestational IH. Collectively, these data indicate that gestational IH impairs energy homeostasis and reduces the number of capillaries per muscle area in the DIA and TA muscles associated with reduced aerobic performance from a younger age. Further analyses are needed to clarify the differences to the responses to gestational IH among the muscles from different body parts.

The present study provides clear evidence regarding the possible adverse effects of gestational IH in the regulation of energy homeostasis and vasculature, at least in part, owing to decreased adiponectin receptor expression in the DIA and TA but not in the GH and MAS muscles of male adolescent offspring rats. The results of the comparison of various skeletal muscle parts are particularly interesting because alterations in energy metabolism by gestational IH depends on the body region. Our findings indicate that a further comprehensive approach to understand the effects of gestational IH on the skeletal muscles of offspring, with consideration of their developmental origins and functions after birth is required.

## Data Availability

The original contributions presented in the study are included in the article/Supplementary Material, further inquiries can be directed to the corresponding author.

## Supplementary material

SUPPLEMENTARY FIGURE S1 Quantification of histological properties of fiber type-dependent NADH activity from NADH-stained sections and glycogen content from PAS-stained sections. **(A)** Representative images of serial sections from the DIA and TA muscles, stained for MHC type I (blue), type IIA (green), type IIX/D (not stained, black) and type IIB (red), NADH-TR, and PAS staining. Bar represents 50 µm. Qualitative analysis of fiber-type-dependent NADH activity in the **(B)** DIA, and **(C)**TA muscles, and of glycogen content from PAS staining in the **(D)** DIA, and **(E)** TA muscles from N/N and IH/N groups. Results are presented as mean ± SD (*n* = 6).

SUPPLEMENTARY FIGURE S2 Western blot images for PPAR-gamma coactivator 1-alpha (PGC1α), mitochondrial transcription factor A (TFAM), NADH:Ubiquinone oxidoreductase complex assembly factor 1 (NDUFAF1), ATP5A1, optic atrophy 1 (OPA1), mitofusin (MFN)1, MFN2, and mitochondrial fission 1 (FIS1) and glyceraldehyde 3-phosphate dehydrogenase (GAPDH) as an endogenous control marker in the **(A)** DIA and **(B)** TA muscles. Quantification of Western blot analysis results of the mitochondrial metabolic markers from the **(C)** DIA and **(D)** TA muscles are shown. The expression level of each protein was normalized to the level of GAPDH expression, and relative expression levels are shown with those for the N/N group set to 1.

SUPPLEMENTARY FIGURE S3 Ratios of CD31-positive capillaries-to-myofiber. The ratios in the DIA (counted around 2,100 capillaries and 1,200 myofibers from each sample), TA (counted around 2,200 capillaries and 1,300 myofibers from each sample), GH (counted around 2,000 capillaries and 1,800 myofibers from each sample), and MAS (counted around 1,900 capillaries and 2,300 myofibers from each sample) muscles were measured from double-stained immunohistochemical images. Results are presented as mean ± SD (*n* = 6) ****p* < 0.001.
