# Corrigendum: Metabolic dysregulation and decreased capillarization in skeletal muscles of male adolescent offspring rats exposed to gestational intermittent hypoxia

**DOI:** 10.3389/fphys.2024.1518152

**Published:** 2024-12-03

**Authors:** Wirongrong Wongkitikamjorn, Eiji Wada, Jun Hosomichi, Hideyuki Maeda, Sirichom Satrawaha, Haixin Hong, Ken-ichi Yoshida, Takashi Ono, Yukiko K. Hayashi

**Affiliations:** ^1^ Department of Orthodontic Science, Graduate School of Medical and Dental Sciences, Tokyo Medical and Dental University (TMDU), Tokyo, Japan; ^2^ Department of Orthodontics, Faculty of Dentistry, Chulalongkorn University, Bangkok, Thailand; ^3^ Department of Pathophysiology, Tokyo Medical University, Tokyo, Japan; ^4^ Department of Forensic Medicine, Tokyo Medical University, Tokyo, Japan; ^5^ Department of Stomatology, Shenzhen University General Hospital, Shenzhen, China

**Keywords:** gestational intermittent hypoxia, skeletal muscle, developmental origins of health and disease (DOHaD), energy metabolism, adiponectin receptors, capillarization

In the published article, there was an error in the sentence that cited the references Gozal et al., 2003 and Camm et al., 2011. A correction has been made to “**4 Discussion**,” paragraph 1, and corrected sentence should appear as: “Subsequently, adult offspring rats exposed to gestational IH gradually increased their body weight (Gozal et al., 2003; Camm et al., 2011).”

The authors apologize for this error and state that this does not change the scientific conclusions of the article in any way. The original article has been updated.

