# Supplementary figures and images for: Metabolic dysregulation and decreased capillarization in skeletal muscles of male adolescent offspring rats exposed to gestational intermittent hypoxia
Source: Front Physiol. 2023 Jan 12;14:1067683. doi: 10.3389/fphys.2023.1067683 (PMC9878705; doi:10.3389/fphys.2023.1067683)

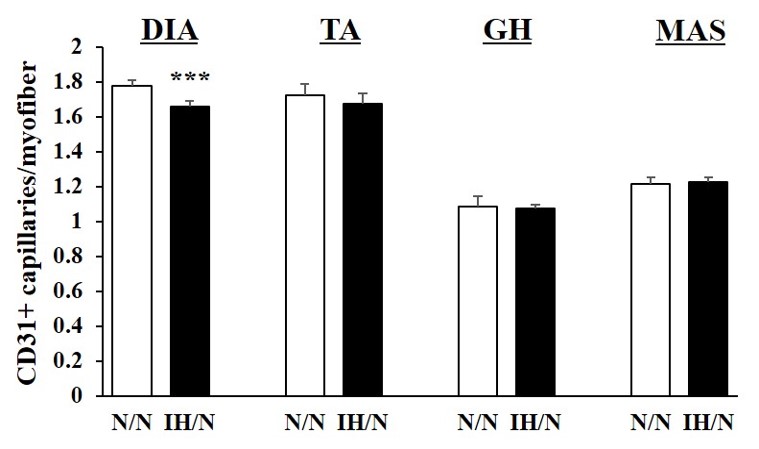

Supplement: Supplementary file 1 [file Image3.JPEG]

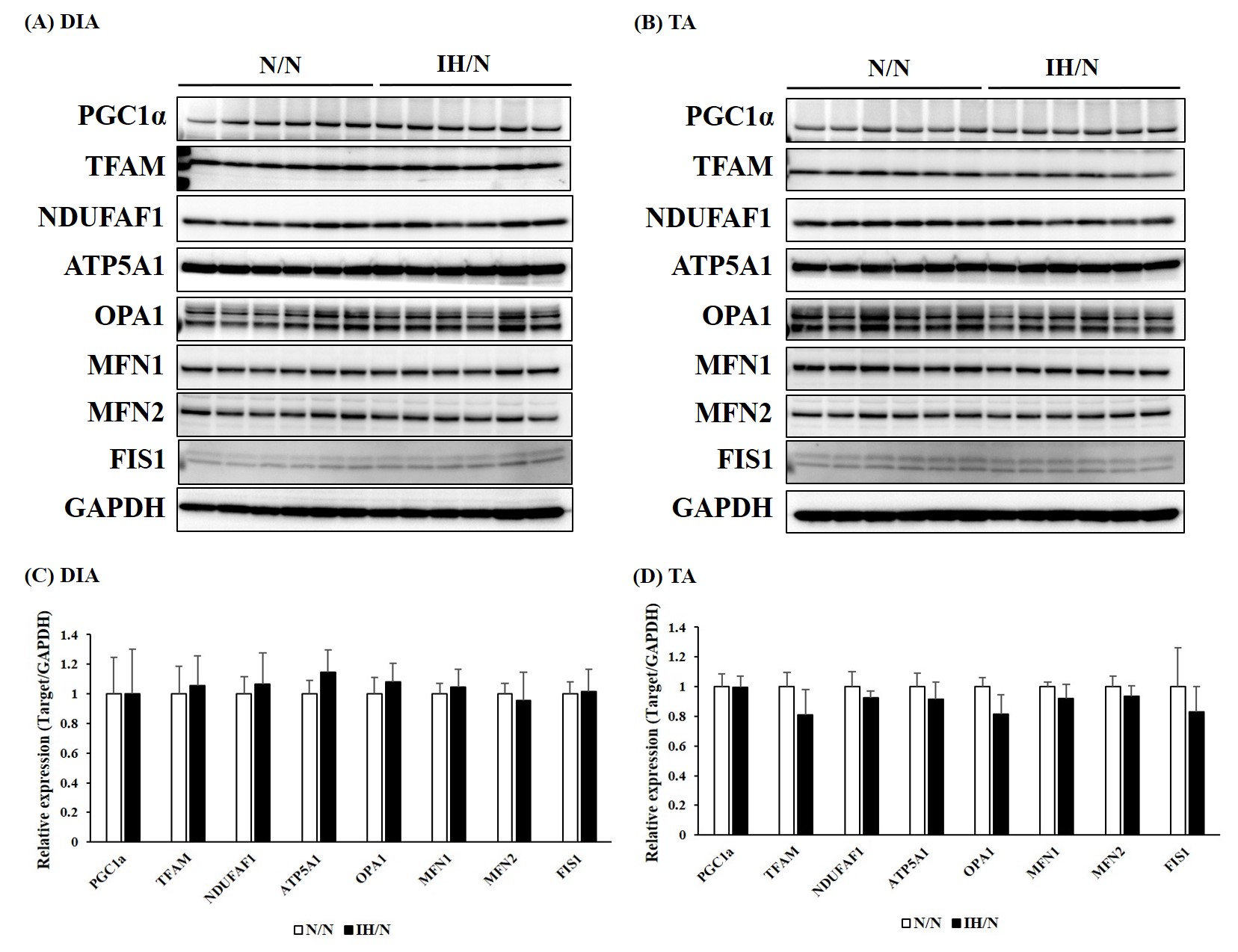

Supplement: Supplementary file 3 [file Image2.JPEG]

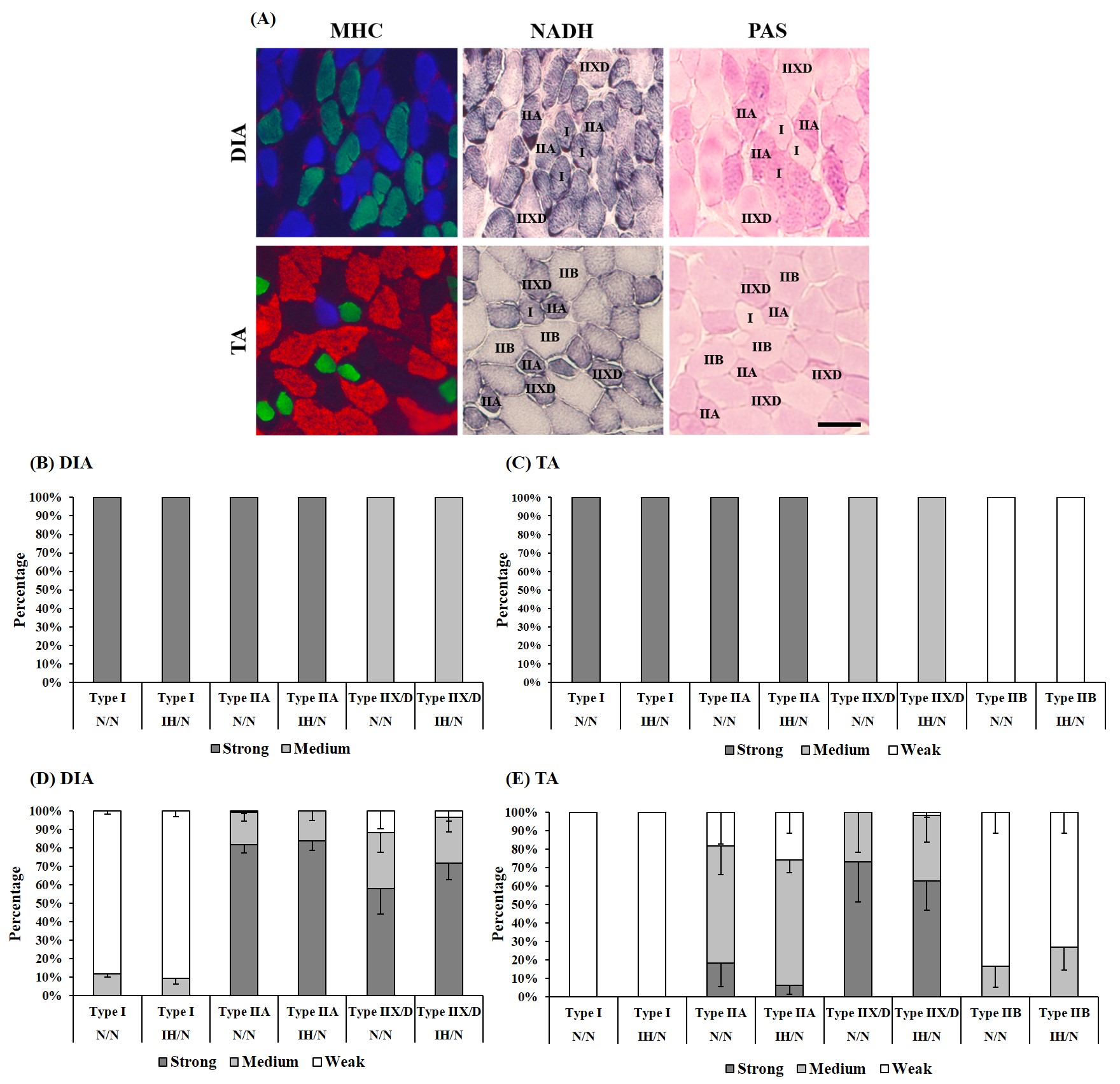

Supplement: Supplementary file 4 [file Image1.jpg]
