# Supplementary material for: Metabolic dysregulation and decreased capillarization in skeletal muscles of male adolescent offspring rats exposed to gestational intermittent hypoxia
Source: Front Physiol. 2023 Jan 12;14:1067683. doi: 10.3389/fphys.2023.1067683 (PMC9878705; doi:10.3389/fphys.2023.1067683)
Supplement: Supplementary file 2 [file Table1.docx]

**Supplemental table 1. List of antibodies used for immunohistochemistry (IHC) and Western blotting (WB)**

| **Antibody** | **Cat. #** | **Vendor** | **Dilution** |
| --- | --- | --- | --- |
| MyHC (type I) | BA-F8 | DSHB | 1:50 (IHC) |
| MyHC (type IIA) | SC-71 | DSHB | 1:600 (IHC) |
| MyHC (type IIX) | 6H1 | DSHB | 1:100 (IHC) |
| MyHC (type IIB) | BF-F3 | DSHB | 1:100 (IHC) |
| Laminin | L9393 | Sigma | 1:500 (IHC) |
| ATP5A1 | ab14748 | Abcam | 1:500 (WB) |
| TTC11 (FIS1) | NB100-56646 | Novus Biologicals | 1:500 (WB) |
| MFN1 | 13798-1-AP | Proteintech | 1:500 (WB) |
| MFN2 | 12186-1-AP | Proteintech | 1:500 (WB) |
| PGC1α | NBP1-04676 | Novus Biologicals | 1:500 (WB) |
| OPA1 | NBP110-55290 | Novus Biologicals | 1:500 (WB) |
| NDUFAF1 | ab79826 | Abcam | 1:500 (WB) |
| TFAM | ab131607 | Abcam | 1:500 (WB) |
| Phospho-mTOR (Ser2448) | 2971 | Cell Signaling | 1:500 (WB) |
| mTOR | 2983 | Cell Signaling | 1:500 (WB) |
| Phospho-PI3 Kinase P85 (Tyr458) | 4228 | Cell Signaling | 1:500 (WB) |
| PI3 Kinase P85 | 4257 | Cell Signaling | 1:500 (WB) |
| PTEN (D3Q6G) | 14642 | Cell Signaling | 1:500 (WB) |
| Phospho-AKT (Ser473) | 9271 | Cell Signaling | 1:500 (WB) |
| AKT | 9272 | Cell Signaling | 1:500 (WB) |
| Phospho-AMPKα | 2531 | Cell Signaling | 1:500 (WB) |
| AMPKα | 2532 | Cell Signaling | 1:500 (WB) |
| HIF1α | ab1 | Abcam | 1:500 (WB) |
| GLUT4 | 66846-1-lg | Proteintech | 1:500 (WB) |
| CD31/PCAM-1 | AF3628 | R&D Systems | 1:500 (IHC) |
| GAPDH | ab8245 | Abcam | 1:10,000 (WB) |

**Supplemental table 2. Primer sequences used for real-time PCR analysis**

| **Gene** | **Forward primer** | **Reverse primer** |
| --- | --- | --- |
| *Pfkm* | 5'-GGGCTGACACAGCACTGAACA-3' | 5'-GGCCAGATAGCCACAGTAACCAC-3' |
| *Pkm* | 5'-TGTTTAGCAGCAGCTTTGATAGTTC-3' | 5'-GCGTGTCACAGCAATGATAGGAG-3' |
| *Pygm* | 5'-TCCGCACACAGCAGCATTACTAC-3' | 5'-TCCAAGGCCAGGTTCACCA-3' |
| *Slc2a1* | 5'-GACCCTGCATCTGATTGGTCTG-3' | 5'-CCACAATGAACCATGGAATAGGA-3' |
| *Slc2a4* | 5'-CTCCAACTGGACCTGTAACTTCATC-3' | 5'-GCCTCTGGTTTCAGGCACTC-3' |
| *Hk1* | 5'-ATTGTCGCCGTGGTGAATGA-3' | 5'-TAGCAAGCATTGGTGCCTGTG-3' |
| *Hk2* | 5'-TCGATGGCTCCGTCTACAAGAA-3' | 5'-ACATCACAGTCGGGCACCAG-3' |
| *Gys1* | 5'-TCAGAGCAAAGCACGAATCCAG-3' | 5'-AACTCATAGCGTCCAGCGATAAAGA-3' |
| *Chrebp* | 5'-AATCCCAGCCCCTACACC-3' | 5'-CTGGGAGGAGCCAATGTG-3' |
| *Lxra* | 5'-CAGGAAGAGATGTCCTTGTGG-3' | 5'-TCTTCCACAACTCCGTTGC-3' |
| *Srebf* | 5'-ACAAGATTGTGGAGCTCAAGG-3' | 5'-TGCGCAAGACAGCAGATTTA-3' |
| *Cpt1* | 5'-CCGAGCTCAGTGAGGACCTA-3' | 5'-ATCTGTTTGAGGGCTTCGTG-3' |
| *Lpl* | 5'-GAACCTGGCCACATCATTTC-3' | 5'-CAGCAAAACCTTTGTGGTGA-3' |
| *Ppara* | 5'-CCTGCCTTCCCTGTGAACT-3' | 5'-ATCTGCTTCAAGTGGGGAGA-3' |
| *Ppargc1b* | 5'-TCTCACACCCCAGTCCAGAA-3' | 5'-AGGCTTGTTGACATCCCGTT-3' |
| *Ppard* | 5'-TGAGTTCTTGCGCAGTATCC-3' | 5'-GCTCCAGAGCATTGAACTTG-3' |
| *Pparg* | 5'-ACCACGGTTGATTTCTCCAG-3' | 5'-CAACCATTGGGTCAGCTCTT-3' |
| *Ucp3* | 5'-GGCCCAACATCACAAGAAAC-3' | 5'-AGCTCCAAAGGCAGAGACAA-3' |
| *Hif1a* | 5'-TCTAGTGAACAGGATGGAATGGA-3' | 5'-TCGTAACTGGTCAGCTGTGGTAA-3' |
| *Adipor1* | 5'-CTTCTACTGCTCCCCACAGC-3' | 5’-TCCCAGGAACACTCCTGCTC-3' |
| *Adipor2* | 5’-TGGGAAGTTTTGTTCCTTGG-3' | 5’-GCAAGGTAGGGATGATTCCA-3' |
| *Actb* | 5'-GGAGATTACTGCCCTGGCTCCTA-3' | 5'-GACTCATCGTACTCCTGCTTGCT-3' |
